# Transcriptomic identification of CREB1 and FOXO1 activation in neuregulin-1-mediated neuroprotection after stroke

**DOI:** 10.3389/fnmol.2026.1741120

**Published:** 2026-03-13

**Authors:** Kimberly R. Bennett, Monique C. Surles-Zeigler, Catherine J. Augello, Etchi Ako, Hakeem Omotayo, Gregory D. Ford, Victor G. J. Rodgers, Byron D. Ford

**Affiliations:** 1 Department of Bioengineering, Bourns College of Engineering, University of California, Riverside, Riverside, CA, United States; 2 Harvard-MIT Division of Health Sciences and Technology, Massachusetts Institute of Technology, Cambridge, MA, United States; 3 Department of Anatomy, Howard University College of Medicine, Washington, DC, United States; 4 Division of Biomedical Sciences, School of Medicine, University of California, Riverside, Riverside, CA, United States

**Keywords:** bioinformatics, ischemic stroke, neuregulin, neuroprotection, transcription factor, transcriptomics

## Abstract

**Introduction:**

Neuregulin-1 (NRG-1) is a growth factor that has been investigated for its neuroprotective properties following ischemic stroke. While NRG-1 has shown considerable promise in reducing neuronal damage, the molecular mechanisms underlying its protective effects remain unclear. This study aimed to examine the impact of NRG-1 treatment on ischemia-induced gene expression following permanent middle cerebral artery occlusion (MCAO) in rats.

**Methods:**

Rats were treated with either NRG-1 or vehicle then sacrificed 3 and 12 h after permanent MCAO. RNA isolated from the peri-infarct cortex (ischemic penumbra) was hybridized to an Affymetrix Rat Genome 2.0 ST Microarray Gene Chip. Gene expression was analyzed using the Affymetrix Transcriptome Analysis Console (TAC) 4.0 software and the STRING Protein–Protein Interaction Networks database.

**Results:**

NRG-1 treatment upregulated transcriptional programs promoting cell survival and anti-inflammatory signaling. CREB1 and FOXO1 transcription factor pathways, which are associated with anti-inflammatory signaling, cell proliferation, reduced apoptosis, and decreased oxidative stress, were upregulated. Consistent with the transcriptomic findings, Luminex multiplex transcription factor assays validated the increased CREB1 and FOXO1 activity in NRG-1-treated MCAO brains.

**Discussion:**

These findings provide novel insight into the molecular mechanisms by which NRG-1 mediates neuroprotection, highlighting its role in activating transcriptional programs that promote neuronal survival and resilience following ischemic injury.

## 1 Introduction

Stroke is a leading cause of death worldwide, with about 87% of cases being ischemic stroke (Fisher et al., 2009; Bosetti et al., 2017; Martin et al., 2025). Unlike hemorrhagic stroke, which results from cerebrovascular bleeding, ischemic stroke occurs when blood flow to the brain is obstructed. This disruption triggers cascades of cell death, inflammation, and oxidative stress, largely mediated by changes in regulatory gene expression (Martin et al., 2025; Jayaraj et al., 2019; Dirnagl et al., 1999; Barone and Feuerstein, 1999). Currently, the only FDA-approved therapy for ischemic stroke is tissue plasminogen activator (tPA), a time-sensitive thrombolytic treatment that restores blood flow. However, tPA is limited to a narrow therapeutic window, and only 3–5% of patients are eligible to receive it (Fisher et al., 2009; Bosetti et al., 2017; Martin et al., 2025). Given the urgent need for more effective neuroprotective therapies, alternative approaches are under investigation.

Following ischemia, the infarct core develops rapidly within minutes, marked by low cerebral blood flow, excitotoxicity, and oxidative stress. This primary injury zone expands over time and triggers the release of pro-inflammatory mediators, which propagate secondary injury. The surrounding ischemic penumbra, by contrast, contains neurons that can remain viable for more than 24 h after stroke onset, extending the potential therapeutic window for intervention (Lipton, 1999).

One promising therapeutic candidate is neuregulin-1 (NRG-1), a small protein initially identified in studies of cancer biology, neuromuscular junction function, and Schwann cell proliferation (Cespedes et al., 2018). Our group and others have shown that exogenous NRG-1 confers neuroprotection in rat models of ischemia by reducing neuronal damage and extending the therapeutic window following middle cerebral artery occlusion (MCAO) (Surles-Zeigler et al., 2018; Xu et al., 2005; Ford et al., 2006; Xu et al., 2004; Rodriguez-Mercado et al., 2012; Simmons et al., 2016; Wang et al., 2015; Xu et al., 2006; Noll et al., 2019; Shyu et al., 2004; Li et al., 2007; Li et al., 2008; Guan et al., 2015). Moreover, NRG-1 has demonstrated potential in promoting post-injury repair after stroke (Iaci et al., 2010; Iaci et al., 2016). Despite these encouraging findings, the molecular mechanisms underlying NRG-1-mediated neuroprotection remain poorly defined.

In this study, we performed a transcriptomic analysis to characterize gene expression changes and transcriptional mechanisms affected by NRG-1 treatment 12 h after permanent MCAO. Using bioinformatics software and protein–protein interaction analysis, we identified key transcription factors regulated by NRG-1. Our findings highlight CREB1 and FOXO1 as mediators of NRG-1-induced neuroprotection. These results provide mechanistic insight into NRG-1’s therapeutic potential and support the rationale for advancing NRG-1 into clinical studies for ischemic stroke.

## 2 Methods

### 2.1 Animals

All procedures were approved by the Institutional Animal Care and Use Committees at Morehouse School of Medicine (Protocol #A-20160020) and the University of California, Riverside (Protocol #20190021) and were conducted in accordance with the ARRIVE guidelines and the AVMA Guidelines for the Euthanasia of Animals. Male adult Sprague–Dawley rats (*Rattus norvegicus*, 250–300 g; Charles River Laboratory International, Inc., United States) were housed in standard cages in a temperature-controlled room (22 ± 2 °C) on a 12-h reverse light–dark cycle with ad libitum access to food and water.

Anesthesia was induced with 5% isoflurane in an O₂/N₂O mixture (30%/70%) inhaled. Core body temperature was monitored via rectal probe and maintained at 37 °C with a homeothermic blanket control unit (Harvard Apparatus, Hollister, MA). Cerebral blood flow (CBF) was continuously monitored with a laser Doppler flowmeter (Perimed, Ardmore, PA), with the probe positioned 7 mm lateral and 2 mm posterior to the bregma in a thinned cranial window. A ≥70% reduction in CBF after occlusion was required for inclusion; all animals survived surgery, and no animals were excluded.

Permanent MCAO (pMCAO) was induced using the intraluminal suture method (Li et al., 2007). Briefly, a 4-0 monofilament nylon suture (4 cm, silicon-coated, Doccol Corp., Sharon, MA) was advanced via the external carotid artery (ECA) into the internal carotid artery (ICA) to permanently occlude the left middle cerebral artery. Rats in the treatment group received 50 μL of recombinant human NRG-1β (20 μg/kg, EGF-like domain, R&D Systems, Minneapolis, MN, 1% BSA in PBS) via bolus injection into the ICA through the ECA immediately prior to MCAO. Vehicle-treated rats received an equivalent injection of 50 μL 1% BSA in PBS. Sham-operated rats underwent identical surgical procedures without insertion of the filament. Animals in the pMCAO and pMCAO + NRG-1 groups were sacrificed 3 (*n* = 4) and 12 h (*n* = 5) after MCAO; sham animals were sacrificed 3 h after surgery (*n* = 4). Euthanasia was performed by overdose with beuthanasia (90 mg/kg injected IP), in compliance with AVMA guidelines. All treatments and analyses were performed in a blinded manner. All animals in this study survived the procedures and none were excluded.

### 2.2 Microarray analysis

Brains were removed immediately after sacrifice and sectioned into 2 mm coronal slices (+3.0 to −3.0 mm from bregma). The section from +1 to −1 mm from bregma was used for RNA isolation while the adjacent section (−1 to −3 mm from bregma) was processed for 2,3,5-triphenyltetrazolium chloride (TTC) staining to verify infarct formation and qualitative reduction by NRG-1 (Li et al., 2007). The microarray analysis was performed on ipsilateral peri-infarct cortical tissue, corresponding primarily to the ischemic penumbra rather than the infarct core. The infarct core tissue was avoided to preserve RNA integrity and to focus on transcriptionally active tissue most relevant to neuroprotection and salvage. In all groups, the total RNA was extracted from ipsilateral cortex using TRIzol Reagent (Life Technologies, Carlsbad, CA) and quality-checked with the Agilent 2100 Bioanalyzer (Agilent Technologies, Santa Clara, CA). RNA was hybridized to the Affymetrix Rat Genome 2.0 ST Gene Chip following the manufacturer’s protocol.

Statistical analysis was performed with Affymetrix Transcriptome Analysis Console (TAC) 4.0. *.CEL files were normalized, quality-checked, and analyzed for differential expression (fold-change), *p*-value and False discovery rate (FDR). Genes were considered differentially expressed at ≥2-fold change with *p* < 0.05 and FDR <0.05. Differential expressed genes encoded transcription factors were annotated using the JASPAR database (Fornes et al., 2020), and their predicted downstream targets were retrieved using the FiserLab TF2DNA database (Pujato et al., 2014). All datasets have been deposited in the NCBI GEO repository (GSE212732).

To identify molecular interactions, differentially expressed genes were analyzed using the STRING database^1^ (Jensen et al., 2009). Each node represents a protein, and edge thickness corresponds to confidence scores derived from co-expression, curated databases, experimental evidence, and association data. A list of NRG-1-upregulated genes (pMCAO vs. pMCAO + NRG-1) was uploaded to STRING to visualize protein–protein interaction networks. Orphan genes without interactors were removed from subsequent analysis. The primary STRING analysis focused on integrated networks across categories to identify transcriptional hubs. Identified transcription factors from JASPAR and confirmed in the transcription factor assay were mapped into STRING, and network expansions were generated to display their first five interactors, highlighting functional clusters and regulatory pathways.

### 2.3 Multiplex transcription factor assay

Ipsilateral cortical tissue adjacent to the microarray sampling site was used (*n* = 3 per condition). Nuclear proteins were isolated with the USB Nuclear Extraction Kit (Affymetrix, Inc.). DNA-binding activity of transcription factors was measured with the Procarta Transcription Factor Assay (Affymetrix, Inc.) according to manufacturer instructions. Protein-DNA complexes were formed by incubating nuclear extracts with Procarta detection probes for 30 min at 15 °C. After a series of cold-binding buffer washes and centrifugation steps, complexes were denatured (95 °C, 5 min) and incubated with capture beads (30 min, 50 °C, 400 rpm). Beads were labeled with streptavidin-PE and read on a Luminex Bio-Plex 200 system (Bio-Rad, Hercules, CA). Activity levels of JASPAR-predicted transcription factors were quantified.

## 3 Results

### 3.1 Temporal gene expression profiles after pMCAO

**Figure 1:**
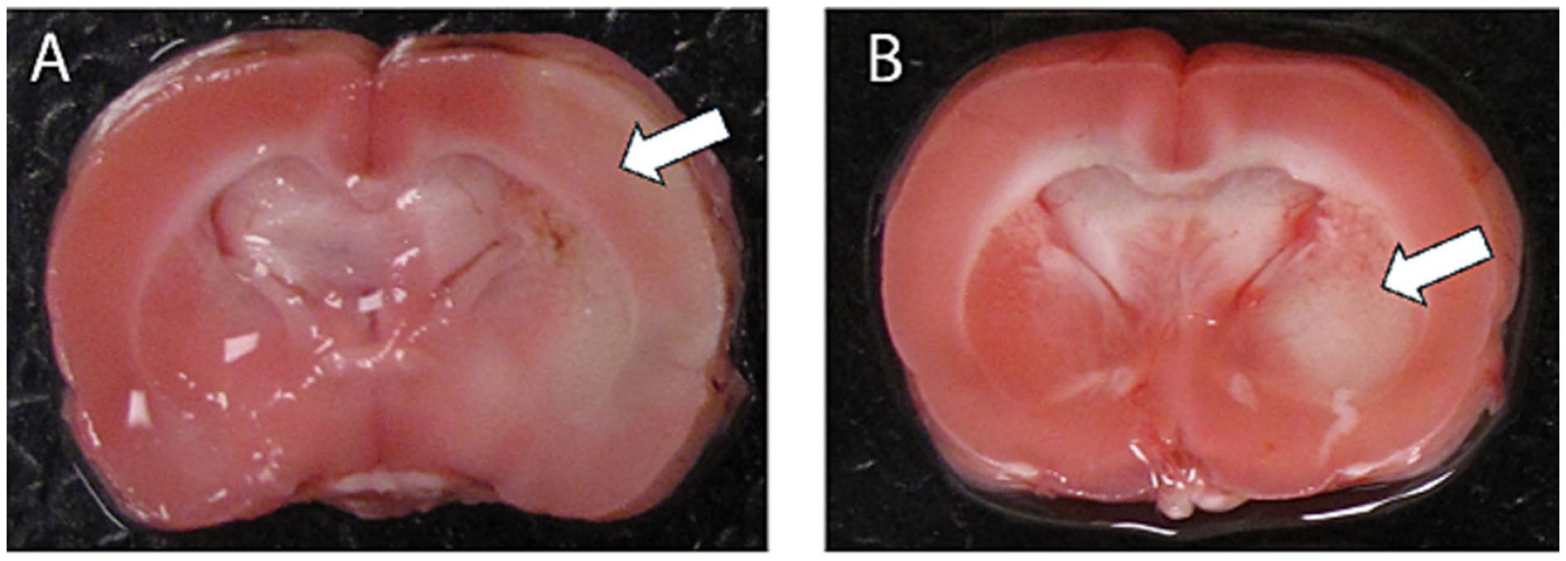
Representative TTC-stained outer coronal sections are shown to confirm infarct formation following MCAO. Sections are not intended for quantitative rostro-caudal comparison, as intermediate tissue was preserved for RNA extraction. TTC staining is provided as qualitative verification of ischemic injury in the cortex of a vehicle-treated animal **(A)** and from NRG-1-treated rats **(B)**. Arrows indicate infarct area.

We previously demonstrated neuroprotection by NRG-1 following transient ischemia in rat models (Surles-Zeigler et al., 2018; Xu et al., 2005; Ford et al., 2006; Xu et al., 2004; Rodriguez-Mercado et al., 2012; Simmons et al., 2016; Wang et al., 2015; Xu et al., 2006; Noll et al., 2019). To investigate early ischemia-induced gene expression changes, rats were treated with NRG-1 or vehicle immediately prior to permanent MCAO. TTC staining at 12 h post-MCAO confirmed substantial infarct formation in cortical and subcortical brain regions in vehicle-treated animals. In NRG-1 treated rats, TTC-stained outer sections suggested a qualitative reduction in infarcted tissue, which was limited to subcortical tissues, relative to vehicle controls (Figure 1). Because intermediate brain sections were preserved for transcriptomic profiling, TTC staining in this cohort was intended as confirmation of ischemic injury rather than quantitative infarct volume assessment. These observations are consistent with prior studies using TTC and MRI that demonstrated infarct-limiting effects of NRG-1 under comparable experimental conditions (Li et al., 2007). Of the 36,685 probes on the Affymetrix Rat 2.0 GeneChip, 10,464 were annotated for the rat genome. Differential expression analysis revealed that 251 genes were significantly altered at 3 h after MCAO (*n* = 4; 230 upregulated, 21 downregulated) and at 12 h after MCAO (*n* = 5; 1,041 upregulated, 1,316 downregulated) compared to sham (Table 1). Thus, both the number of upregulated and downregulated genes progressively increased with time after MCAO. Analysis of genes regulated by stroke after 3 h was previously described (Surles-Zeigler et al., 2018) and since differential expression was most pronounced at 12 h, this timepoint was selected for further analysis.

**Table 1:** Genes up- and downregulated 2-fold or more 3 and 12 h following MCAO vs. sham.

Comparison	Upregulated	Downregulated
3 h MCAO vs. sham	230	21
12 h MCAO vs. sham	1,041	1,316

### 3.2 Transcriptomic changes following MCAO and NRG-1 treatment

**Figure 2:**
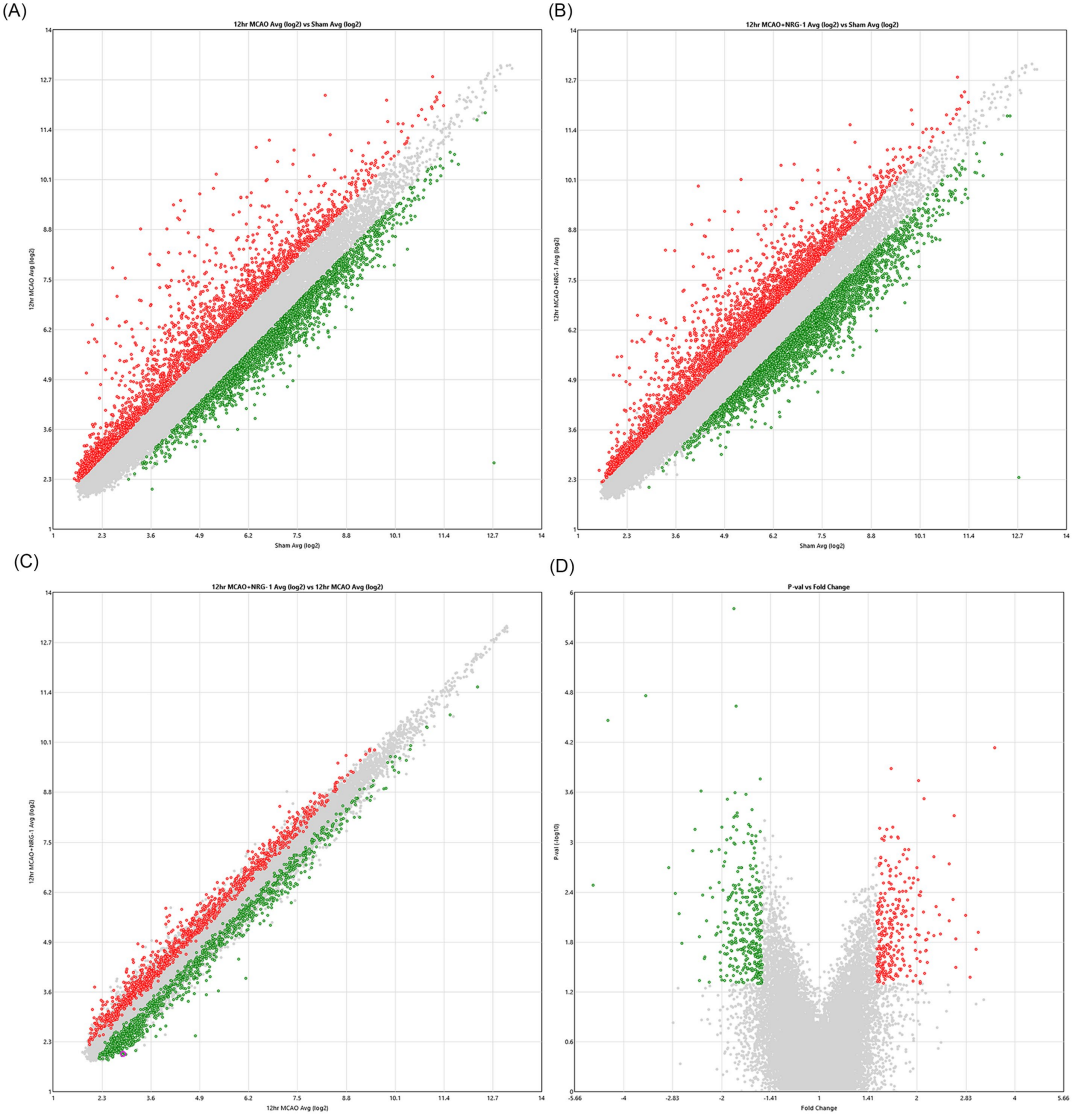
Differential gene expression analysis. **(A)** 2,357 genes differentially expressed between sham and MCAO. **(B)** 2,348 genes differentially expressed between sham and MCAO + NRG-1. **(C)** 635,253 genes were differentially expressed between MCAO and MCAO + NRG-1, highlighting NRG-1-mediated regulation (253 upregulated, 382 downregulated). **(D)** Volcano plot showing significance versus fold-change distribution. Red dots (upper left side) indicate genes that were upregulated between these conditions and green dots (lower right side) indicate genes that were downregulated.

Microarray scatter and volcano plots illustrate the distribution of differentially expressed genes across conditions. Compared to sham, pMCAO alone altered 2,357 genes expressed (Figure 2A). The sham versus MCAO + NRG-1 comparison revealed 2,348 differentially expressed genes (Figure 2B). Importantly, when directly comparing pMCAO + vehicle to pMCAO + NRG-1, 635 genes were differentially expressed between MCAO and MCAO + NRG-1 (253 upregulated; 382 downregulated) (Figures 2C,D). The differential upregulated genes were prioritized for subsequent downstream pathway analysis. While both up- and down-regulated genes are biologically informative, downstream analyses were intentionally restricted to NRG-1-upregulated transcripts to prioritize identification of pro-survival and anti-inflammatory signaling networks, which represented the central mechanistic focus of this study. Additional studies will be required to determine whether the observed downregulation reflects active transcriptional repression or instead results from the loss of specific cell populations that normally express those transcripts.

### 3.3 Functional clustering of NRG-1-regulated genes

To explore functional patterns, the 253 differentially upregulated expressed genes were grouped into three categories (Table 2). Functional groupings in Table 2 were based on Gene Ontology pathway annotation (TAC functional categories) and STRING enrichment patterns. Genes upregulated by pMCAO and further increased by NRG-1 (*n* = 26) were associated with metabolic processes, cellular growth, biological regulation, and inflammatory signaling. Genes unchanged by MCAO but upregulated by NRG-1 (*n* = 188) were linked to oxidative stress response, DNA replication, PI3K signaling, and growth factor pathways. Notably, this group included transcription factors CREB1, HIC2, and ZBTB26. Genes downregulated by MCAO but rescued by NRG-1 (*n* = 39) were enriched in pathways regulating cellular development, metabolic function, and PI3K/Wnt signaling. Transcription factors ZIC2 and FOXO1 were identified in this cluster.

**Table 2:** Genes with change following MCAO and subsequent regulation by NRG-1.

Gene expression changes	Gene (*n*)	Gene symbols
Genes increased with MCAO (fc > 1.5) and further increase with NRG-1 (fc > 1.5)	26	Tomm70a, Tcea1, Ppwd1, Eed, Tceal8, Thoc7, Nipbl, Sult1d1, Zfp62, Swt1, Hnrnpm, Plcxd3, Actr6, Chpt1, Ppp4r3b, Fam135a, Btg3, Ccdc88a, Il18, Cep290, Rock1, Zfp780b, Orc4, Prpf39, Nop58, Nts
Genes maintained following MCAO (1.5 > fc > −1.5) and increased with NRG-1 (fc > 1.5)	188	Mir423, Efcab1, Ccdc171, Pros1, Cbx3, Ostc, Smarca1, Rbm34, Tc2n, Nap1l5, Bex4, Dppa3l1, Mum1l1, Mier1, Xrn1, Sirt1, Suv420h1, Apoo, Actl6a, Ube2d2, Bag4, Zfand4, Morf4l1, Cspp1, Rbbp8, Vom1r7, Zfp458, Diaph2, Spag6, Hgf, Hipk3, Olr1601, Olr1257, Olr1736, Morc4, Dnajc24, Lpl, Plek2, Phip, Dmxl1, Dzip3, Lrif1, Vcsa1, Mgat4c, Casp12, Ssb, Mipol1, Spata5, Tatdn1, Tmem251, Olr1214, Lrch2, Slc5a7, Erlec1, Zfp605, Creb1, Ireb2, Stag1, U2surp, Trnt1, Rasa2, Lrrtm2, Kcna4, Calu, Zfp280c, Uba6, Serpinb6, Chmp5, Zfp638, Nbeal1, Pbdc1, Nae1, Senp6, Cep57, March7, Apool, Trpm7, Dsel, Dek, Syt10, Zfp280d, Zfp945, Rsbn1, Exoc5, Prpf40a, Cul5, Mpp6, Luc7l3, Micu3 Plppr1, Rnf223, Znf354b, Unc13d, Rnf113a1, Hsd17b8, Psx1, Rassf7, Adora2b, Ddx59, Sdsl, Nek10, Mboat2, Nap1l2, Slc25a45, Olr1488, Cox19, Zfp420, Prim1, Npy2r, Lrrcc1, Cyp11b2, Kpna7, Hnmt, Slx4ip, Nfkbil1, Ubxn2b, Kazald1, Rnase4, Mei4, Tfdp2, Mcts2, Slc6a18, Strip2, Rpl26, Npy2r, Pus10, Slc25a2, Hic2, Ankdd1b, Lrrn4cl, Fcho2, Ccdc191, Xrcc2, Fbn2, Ces2e, Olr87, Dusp13, Olr1462, Scaper, Slc18a2, Cdca7l, Rbmxl1, Ghr, Timm10b, Olr860, Topors, Xpa, Zfp597, Eci3, Jrkl, Olr777, Ifit1bl, Ptges3l1, St8sia4, Kif4b, Fsd1l, Epha6, Gabpb1l, Il1rapl1, Gk, Pik3r3, Pde1c, Ccdc69, Gabpb1, Ints8, Dock11, Fam133b, Vom1r19, Acp1, Zbtb26, Kctd4, Ly6i, Ccl28, Atp6ap1l, Cenpk, Rc3h1, Rnf128, Pola1, Pts, Csnk1g3, Kif24, Mir181b2, Usp37, Cyp7b1, Snx24, Cmpk1, Prkar2b, Tceb1
Genes decreased following MCAO (fc < −1.5) and increased with NRG-1 (fc > 1.5)	39	Igkv5-48, Zic2, Cnnm3, Mrpl34, Sf3a2, Ccdc141, Pcdh19, Ebf4, Rwdd3, Eda, Ep300, Olr1313, Cpt1b, Elac1, Pcdhb9, Cdhr1, Pdcl, Foxo1, Dmrtc1b, Smim17, Mir181c, Tmem126b, Jade1, Ndst4, Obp2b, Mir21, Atp6v0a2, Fancg, Accs, Hsd11b2, Zfp867, Echdc2, Kdelc2, Pfkfb2, Slc45a2, Rpgrip1l, Adprhl1, Snapc5, Rslcan18

### 3.4 Network analysis reveals transcriptional hubs

**Figure 3:**
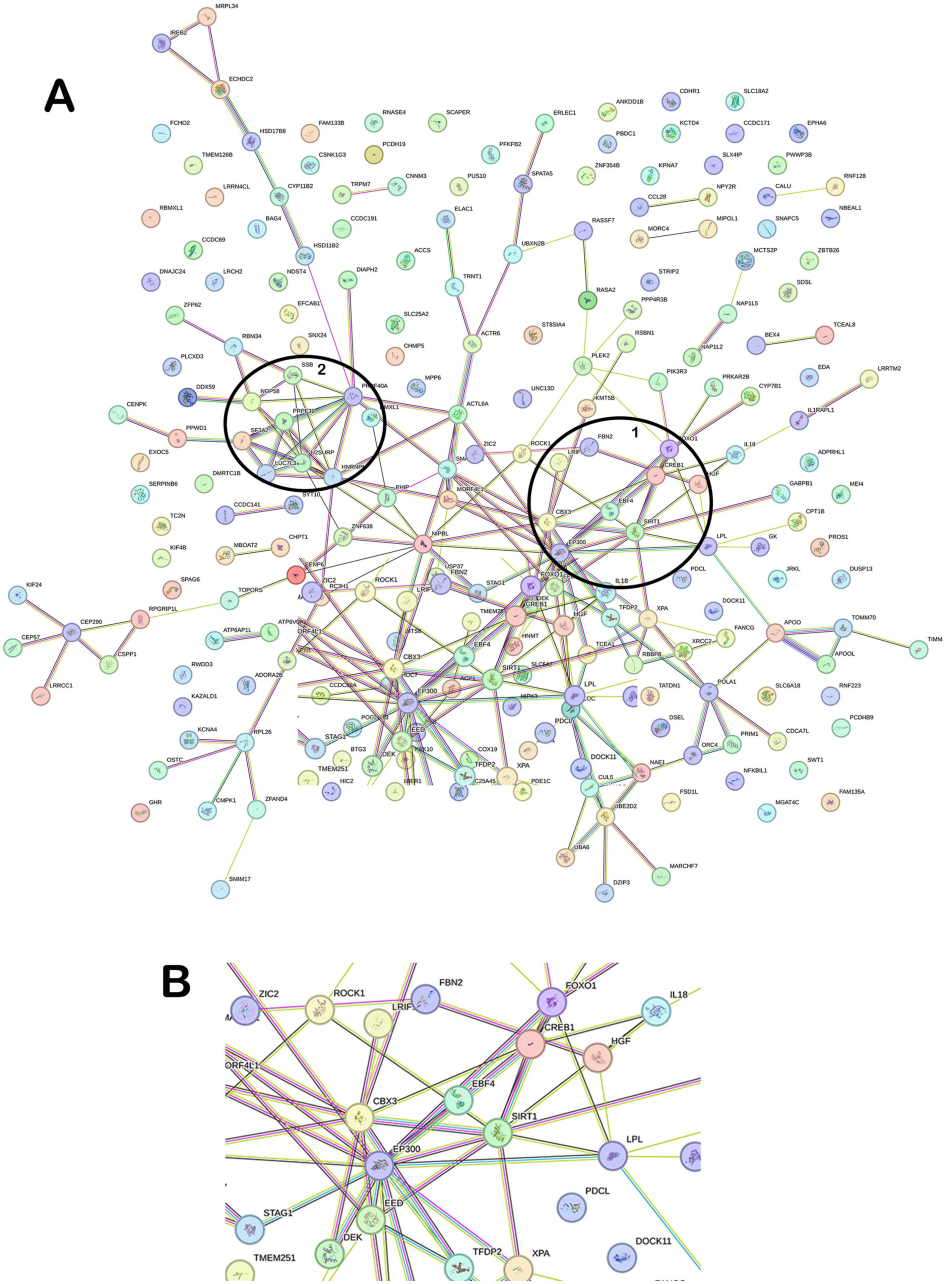
STRING network analysis of NRG-1–upregulated genes (*n* = 253). **(A)** Network analysis identified two major interaction hubs. Hub 1 features CREB1 and FOXO1 as central transcriptional regulators, while Hub 2 represents a second densely connected cluster. Genes without known interactions (orphans) were excluded from the network. **(B)** Enlarged view of Hub 1 highlighting its core regulatory components.

The 253 NRG-1–responsive genes in Table 2 were analyzed using STRING. Protein-protein interactions visualized using STRING are used to identify potential “hub” genes that signify regulatory factors and related pathways. Each connecting line represents a known or predicted protein interaction. Of the 5 transcription factors identified in the microarray analysis, only CREB1, FOXO1, and ZBTB26 were connected to the network. Two major hubs were identified, with Hub 1 containing the transcription factors CREB1 and FOXO1, both tightly connected in the network (Figure 3A). Hub 2 represented peripheral interaction groups with less central regulatory influence and limited known neuroprotective relevance. Because of their centrality and known roles in neuroprotection, Hub 1 was prioritized for further study (Figures 3A,B).

### 3.5 CREB1 and FOXO1 downstream targets

**Figure 4:**
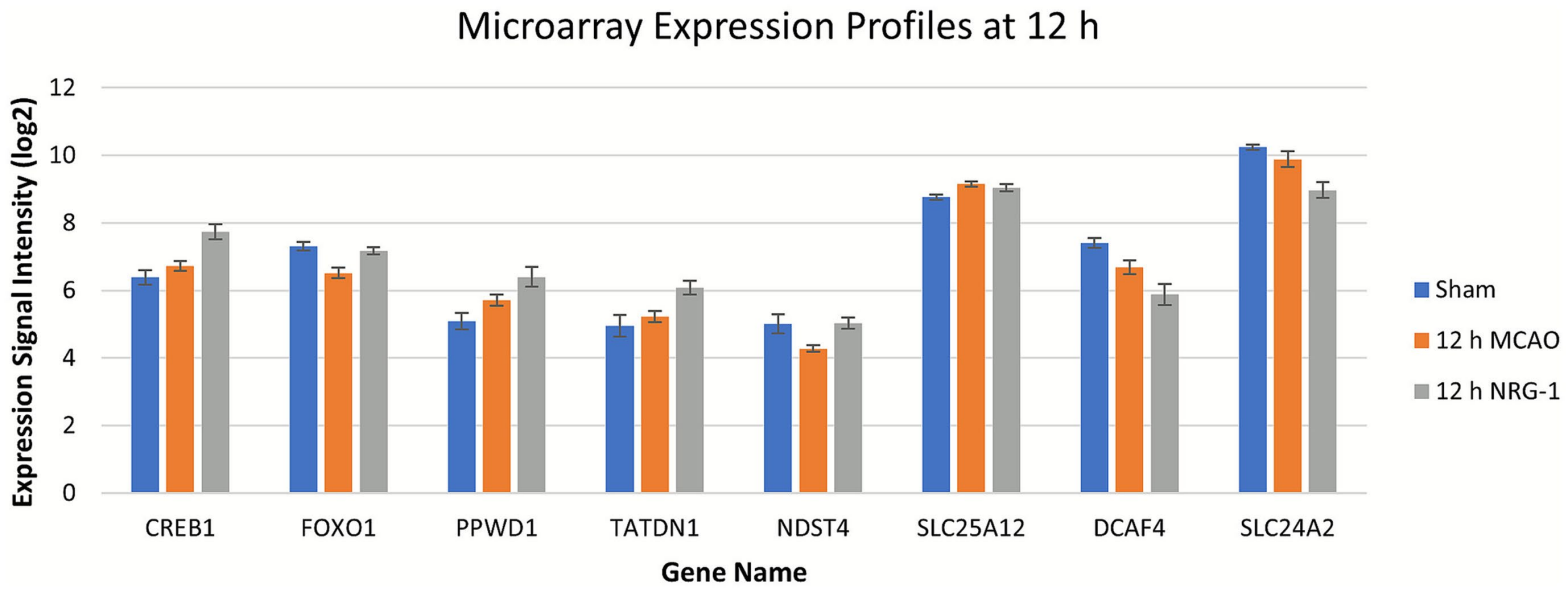
Expression profiles of CREB1, FOXO1, and their downstream targets. NRG-1 upregulated CREB1 and FOXO1 expression and modulated several downstream targets, including PPWD1, TATDN1, NDST4, SLC25A12, DCAF4, and SLC24A2.

To probe functional relevance, we examined known CREB1 and FOXO1 targets using the FiserLab TF2DNA database. Several targets overlapped with the expressed genes were present in our dataset. For example, FOXO1 and its target NDST4 were downregulated by MCAO and rescued with NRG-1, while CREB1 and its targets PPWD1 and TATDN1 showed further upregulation after NRG-1. These findings suggest that NRG-1 enhances pro-survival transcriptional programs via CREB1 and FOXO1 (Figure 4).

### 3.6 Pathway integration with PI3K-AKT signaling

**Figure 5:**
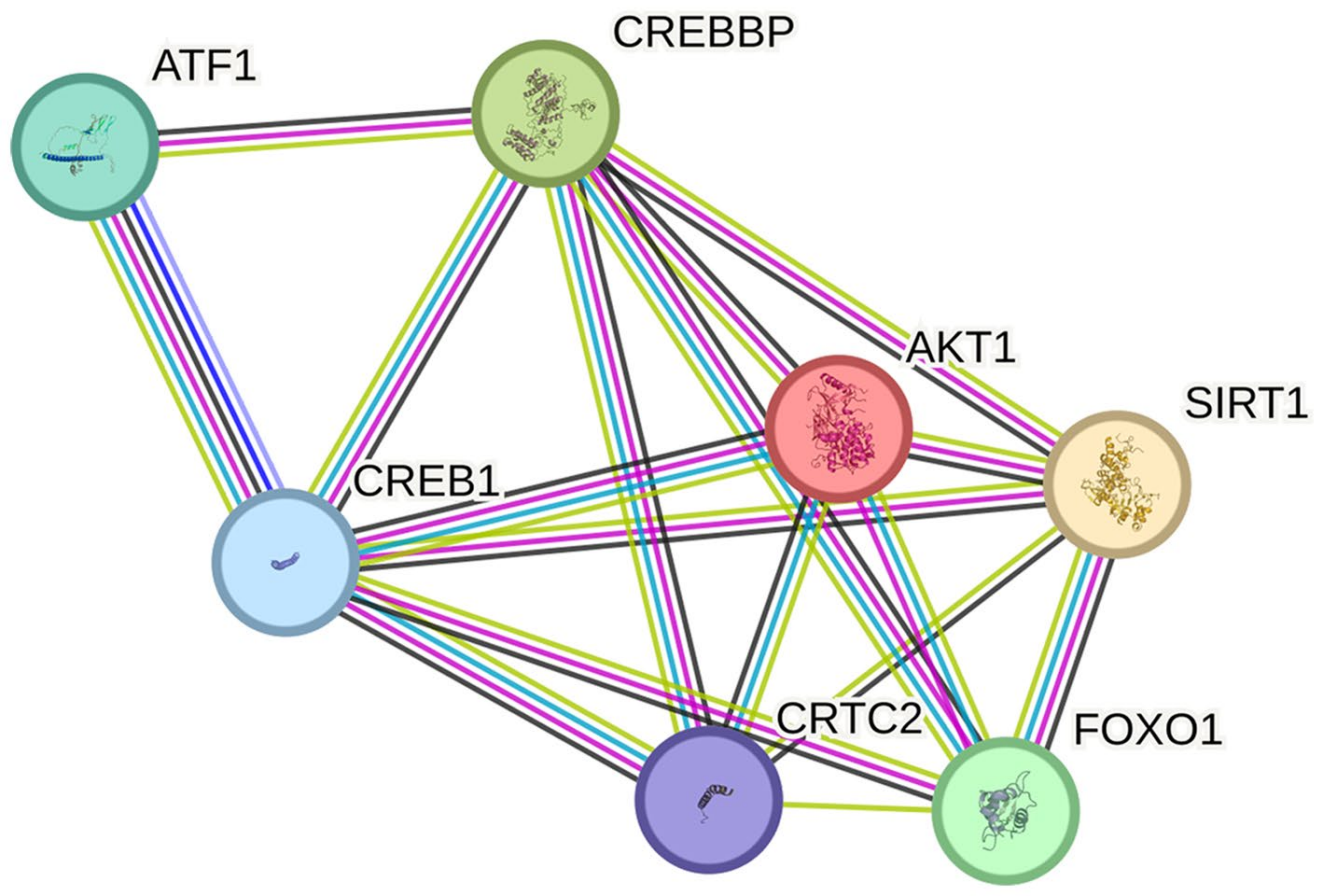
Expanded STRING network of CREB1 and FOXO1, showing five interactors (CREBBP, SIRT1, CRTC2, AKT1, ATF1). SIRT1 was upregulated by NRG-1. Many interactors are part of the PI3K-AKT survival pathway.

STRING expansion of CREB1 and FOXO1 revealed high-confidence interactions with CREBBP, SIRT1, CRTC2, AKT1, and ATF1. Among these, SIRT1 was significantly upregulated by NRG-1 in our dataset compared to pMCAO and vehicle controls. Several interactors participate in the PI3K-AKT pathway, a key mediator of cell survival, highlighting a potential mechanism by which NRG-1 promotes neuroprotection (Figure 5).

### 3.7 Multiplex transcription factor array

**Figure 6:**
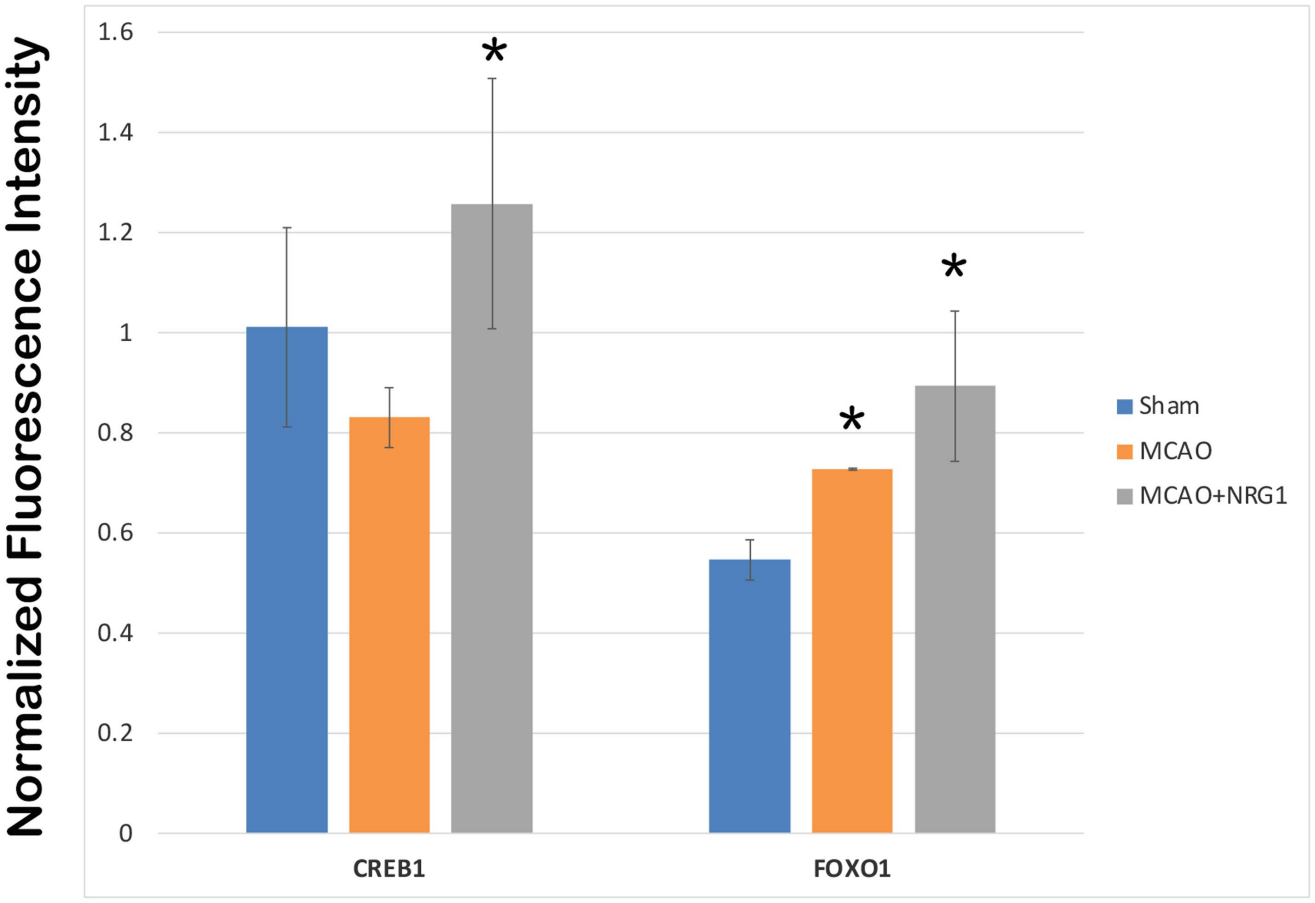
Multiplex transcription factor assay. CREB1 was not altered after MCAO, but was significantly after MCAO and NRG-1 treatment. FOXO1 activity was increased by MCAO and further enhanced by NRG-1 treatment. ^*^Denotes significantly increased compared to sham control.

To validate that transcriptomic changes in CREB1 and FOXO1 corresponded with activity, a multiplex transcription factor assay was performed on nuclear extracts from the brains of sham, pMCAO, and pMCAO + NRG1 animals. CREB1 was not altered after MCAO, but was significantly after MCAO and NRG-1 treatment (Figure 6). FOXO1 activity was increased by MCAO and further enhanced by NRG-1 treatment. These findings suggest that CREB1 and FOXO1 regulation and increased activity following NRG-1 treatment are a potential mechanism of neuroprotection worth further exploration.

## 4 Discussion

NRG-1 has demonstrated neuroprotective and anti-inflammatory effects in rodent models of ischemic stroke (Surles-Zeigler et al., 2018; Xu et al., 2005; Ford et al., 2006; Xu et al., 2004; Rodriguez-Mercado et al., 2012; Simmons et al., 2016; Wang et al., 2015; Xu et al., 2006; Noll et al., 2019). However, the molecular mechanisms underlying these effects remain incompletely understood. We previously showed that NRG-1 mediates its protective effects through activation of the PI3K-Akt pathway and inhibition of canonical NF-κB signaling (Simmons et al., 2016). The present study extends these findings by integrating microarray and transcription factor array analyses from infarct tissue 12 h after stroke, revealing novel insights into the transcriptional networks regulated by NRG-1. The rationale for administering NRG-1 immediately prior to pMCAO was to maximize mechanistic resolution during the early ischemic phase in the pMCAO model, consistent with our prior transcriptomic and neuroprotection studies. We acknowledge that pre-treatment limits immediate clinical translatability. However, we have shown that NRG-1 is protective when administered hours after MCAO (Xu et al., 2006).

In this study, we validated the functional relevance of key transcriptomic findings using multiplex transcription factor activity assays, rather than individual gene expression. CREB1 and FOXO1 emerged as potential key regulators of NRG-1-mediated effects. These transcription factors are known to play pivotal roles in neuronal survival, and their functions appear to be interconnected and mutually dependent. CREB1, activated via the co-activator EP300, regulates pro-survival gene expression and suppresses NF-κB-mediated inflammation. Its activation by NRG-1 in this study aligns with known CREB1 roles in reducing apoptosis and promoting neuronal survival after ischemia (Wen et al., 2010; Zhang et al., 2020; Mohamed et al., 2019; Bai et al., 2018). Our findings support a role for CREB1 in NRG-1-mediated neuroprotection. Specifically, ep300 upregulation suggests CREB activation, consistent with prior work showing that NRG-1 alone increases CREB expression in the absence of MCAO. This is in line with our *in vitro* results showing NRG-1 suppresses NF-κB-mediated inflammatory responses (Simmons et al., 2016). Collectively, these data suggest that NRG-1 primes neuroprotective transcriptional pathways to counteract ischemic injury.

The forkhead box O (FOXO) family of transcription factors has been implicated in multiple neurological disorders, including Alzheimer’s disease, Parkinson’s disease, and amyotrophic lateral sclerosis (Maiese, 2015; Maiese, 2021). FOXO1 and FOXO3 regulate apoptosis, oxidative stress, and autophagy downstream of Akt and SIRT1. Importantly, estradiol has been shown to preserve FOXO1 phosphorylation and prevent neuronal death after ischemia (Jover-Mengual et al., 2010). Similarly, FOXO1 upregulation by NRG-1 supports a complementary role in reducing oxidative stress and apoptosis, acting alongside CREB1 to promote neuronal survival after ischemia.

We also observed increased expression of SIRT1, a NAD^+^-dependent histone deacetylase downstream of PI3K-Akt signaling. SIRT1 regulates apoptosis, stress resistance, and longevity by deacetylating targets such as p53, NF-κB, and FOXO proteins (Chae and Broxmeyer, 2011). Previous studies have shown that SIRT1 promotes FOXO1-induced autophagy, reduces apoptosis, and protects against ischemia/hypoxia (Meng et al., 2017; Chen et al., 2009; Yeung et al., 2004; Brunet et al., 2004; Hattori et al., 2015). Our results are consistent with these findings, suggesting that NRG-1-mediated SIRT1 upregulation may enhance FOXO1 activity while concurrently suppressing NF-κB signaling.

The present findings complement our previous transcriptomic study, which identified ETS1 as a key transcriptional regulator of NRG-1-mediated gene expression following ischemic stroke (Surles-Zeigler et al., 2018). While ETS1 was primarily associated with modulation of inflammatory and stress-response genes, the current data indicate that CREB1 and FOXO1 govern a distinct but intersecting network focused on cell survival, oxidative stress resistance, and metabolic regulation. Together, these studies suggest that NRG-1 engages a coordinated transcriptional program involving both ETS1-driven repression of pro-inflammatory cascades and CREB1/FOXO1-driven activation of pro-survival signaling. This integrated mechanism highlights how NRG-1 exerts multifaceted neuroprotective effects, attenuating inflammation while simultaneously promoting neuronal resilience and repair.

In conclusion, this study suggests CREB1 and FOXO1 are key mediators of NRG-1-mediated neuroprotection in ischemic stroke. We acknowledge that NRG-1-mediated transcriptional programs likely differ depending on treatment timing and stage of injury, and that CREB1/FOXO1 regulation may represent one of several temporally distinct protective mechanisms. Future work will assess post-treatment transcriptomes in permanent and transient stroke models. These findings expand upon prior work implicating PI3K-Akt signaling and NF-κB inhibition in NRG-1 action. A limitation of this study is the requirement for pre-treatment with NRG-1 in the pMCAO model. However, we and others have demonstrated that NRG-1 remains protective when administered up to 13.5 h after transient MCAO, supporting a clinically relevant therapeutic window. We further propose that NRG-1 may have utility as a prophylactic treatment in settings such as cardiac surgery or subarachnoid hemorrhage, where ischemic brain injury is a known risk.

An important limitation of the present study is that all experiments were performed in adult male rats, which may not fully capture the influence of sex- or age-related biological variability on NRG-1-mediated neuroprotection. Sex differences in stroke pathophysiology and treatment response are well-documented, with hormonal and genetic factors influencing neuronal survival, inflammation, and recovery trajectories. Although the current data demonstrate NRG-1 efficacy in male rats, our previous studies established that NRG-1 provides comparable neuroprotection in both male and female mice following focal ischemic stroke, suggesting that its protective mechanisms may be broadly conserved across sexes (Noll et al., 2019). Nevertheless, future studies should explicitly evaluate sex- and age-dependent effects of NRG-1 treatment and downstream CREB1/FOXO1 activation to ensure translational relevance, particularly given the increased stroke incidence and altered neuroinflammatory responses observed in aged and post-menopausal populations.

We also acknowledge that TTC staining was performed on the two outer coronal sections to preserve peri-infarct cortex for transcriptomic analysis. Accordingly, TTC staining served to confirm infarct formation and suggest a qualitative reduction with NRG-1, rather than providing statistically quantified infarct volume comparisons. We therefore reference our prior quantitative MRI and TTC studies using the same dosing and delivery paradigm which demonstrated reproducible neuroprotective effects of NRG-1 in the pMCAO model (Ford et al., 2006; Wang et al., 2015; Li et al., 2007). Because behavioral deficits and recovery trajectories typically evolve over days to weeks after MCAO, comprehensive behavioral testing falls outside the scope of this acute molecular profiling dataset. Nevertheless, our prior studies also demonstrated that NRG-1 significantly improves neurological and functional outcomes following MCAO. Future work will integrate transcriptional network findings with long-term functional recovery studies.

In addition, we recognize that inclusion of a sham + NRG-1 group would further help distinguish transcriptional effects attributable to NRG-1 alone from those specific to ischemic injury. However, the present experimental design was focused on identifying NRG-1 dependent modulation of ischemia-induced transcriptional responses within the peri-infarct region, rather than baseline effects of NRG-1 in uninjured brain. Future studies will systematically evaluate NRG-1 only effects across brain regions and treatment conditions.

The identification of CREB1 and FOXO1 as downstream effectors of NRG-1 signaling provides new opportunities for translational development. Modulation of these transcription factors could serve as a mechanistic framework for next-generation NRG-1-based therapeutics. For instance, small molecules or peptide mimetics designed to enhance CREB1 phosphorylation or FOXO1 nuclear translocation could replicate the pro-survival and anti-inflammatory benefits of NRG-1 without requiring direct receptor engagement. Such agents might act synergistically with PI3K-Akt or SIRT1 activators, amplifying cellular resilience to oxidative and ischemic stress. Furthermore, combinational strategies incorporating NRG-1 analogs with CREB1/FOXO1 co-agonists may extend the therapeutic window for ischemic stroke and reduce long-term neuroinflammation. Given that NRG-1 derivatives like cimaglermin are already in clinical trials for cardiac repair, mechanistic targeting of the CREB1-FOXO1 axis could accelerate repurposing efforts for neuroprotection and inform biomarker-guided precision medicine approaches in acute stroke management.

**Figure 7:**
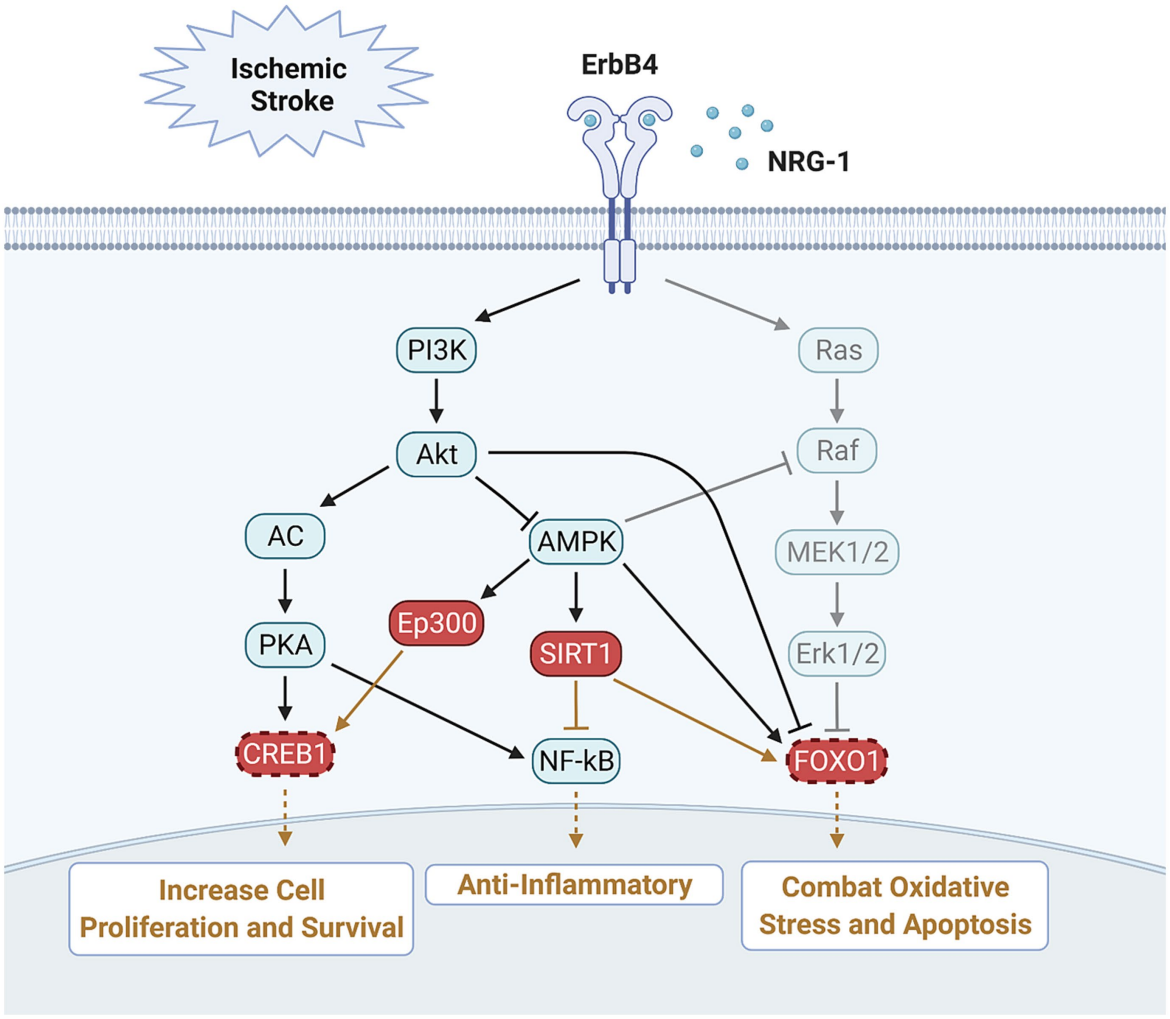
The model illustrates proposed molecular mechanisms for how NRG-1 prevents neuronal death and neuroinflammation following ischemic stroke.

Based on our findings, we propose a model in which NRG-1 engages erbB4 receptors to activate PI3K-Akt signaling, leading to CREB1 activation and enhanced neuronal survival. Simultaneously, FOXO1 upregulation reduces oxidative stress and apoptosis, while SIRT1 activation inhibits NF-κB-mediated inflammation. Collectively, these mechanisms converge to promote neuroprotection and reduce neuroinflammation following ischemic stroke (Figure 7). NRG-1 isoforms are already in clinical trials for heart failure and have shown benefit in improving cardiac function (Meyer and Birchmeier, 1995). Our findings suggest that NRG-1 or NRG-1-based therapeutics could be repurposed for ischemic stroke, offering both acute neuroprotection and long-term therapeutic potential.

## Data Availability

The datasets presented in this study can be found in online repositories. The names of the repository/repositories and accession number(s) can be found below: https://www.ncbi.nlm.nih.gov/, GSE212732.

## Footnotes

1
https://string-db.org
